# The Power of Adaptation to Environment as Shown by Cells and Tissues in Various Pathological Processes

**Published:** 1899-06

**Authors:** Frank A. Delabarre

**Affiliations:** Boston, Mass.


### THE POWER OF ADAPTATION TO ENVIRONMENT AS SHOWN BY CELLS AND TISSUES IN VARIOUS PATHOLOGICAL PROCESSES.[1]

BY FRANK A. DELABARRE, D.D.S., M.D., BOSTON, MASS.

The wonderful mechanism of the human body, with its intricate and interdependent machinery nourished and actuated to motion by the delicate processes of physiological action of the different systems, is a marvellous thing. The manifestations of physiological activity lose half their impressiveness in their very familiarity. Anatomy, to the layman particularly, does not seem very wonderful because the functions of the different muscles and organs are so commonplace. We breathe and think nothing of it. Our heart keeps on beating day and night, year in and year out, through all the varying mental emotions that engross our attention. The nervous system, the process of assimilation, distribution, and excretion, and the circulatory system, with its tremendous network of veins and arteries, all reveal the wonderful power and perfection exemplified in their interdependent and harmonious work.

Pathology introduces abnormal conditions and imposes unusual demands upon the system, stimulating the cells or organs to increased activity or diminishing their normal action. Under such circumstances is shown the power of adaptation to environment, the compensatory action striving to preserve the balance at the normal physiological point.

Man can build a locomotive with intricate machinery and marvellous power, and yet he cannot instil into that product of his genius the ability to repair a weak spot in the boiler or to mend a broken crank-pin. It is this attribute of nature that is even more surprising than the physiological functions. The fact that nature can recognize the existence of an abnormal condition and then take steps to eliminate it, or, failing in that, to adapt herself to it, is the central idea that is emphasized in this paper.

Inflammation is the accompaniment of almost all pathological processes, and to it one must look for an explanation of the methods

---

[1] Read before the American Academy of Dental Science, Boston, January 4, 1899.

nature pursues in effecting a partial or complete restoration to normal conditions when circumstances impair the usefulness of any part.

To define it, "Inflammation is the phagocytic reaction of the organism to the action of an irritant." This power of enveloping and disposing of foreign bodies is possessed by the leucocytes, all connective tissue cells, and some epithelial cells. These cells are the active ones in combating disease and injury.

Irritation being present, there first occurs a change in the vascular system. After a momentary contraction there is a dilatation of the blood-vessels with slowing of the current and passage of leucocytes through the walls, followed by a diapedesis of the red blood-corpuscles and an exudate of plasma through the enlarged stomata. The connective tissue cells and slumbering cells migrate to the scene of activity and proliferate. The chemotactic action of these cells is directed against foreign bodies and, as their power is less or greater than the action of the irritant, degeneration or regeneration occurs.

Degeneration results in destruction of the part or whole, and its process does not come under consideration. Regeneration is accomplished either by resolution, or the absorption of the exudate by the lymphatics and dissolution of the irritant, or by suppuration,—if the lesion is an aggravated one,—when the effete matter is thrown off in the form of pus. If these means fail, organization or the permanent isolation of the irritant is attempted. Cicatrization follows with the evolution of round connective tissue cells into new tissue and the development of a new circulatory system and repair of the nerve-fibres.

Turning now to a consideration of some of the few examples among the many that could be cited on this subject as peculiarly showing this latent power of nature, take first the action of the lachrymal glands when the eye is irritated. The normal secretion is tremendously increased, which, with the involuntary movements of the lid, tends to move the foreign body towards the inner canthus and off from the sensitive cornea.

When, by caries, fracture, or abrasion, the dental pulp becomes irritated by thermal changes or acid secretions, nervous impulses start the odontoblasts to making a deposition of additional layers of dentine to protect the vital portion of the tooth.

Irritation set up by moving the teeth induces an absorption of the bony alveolus by the osteoclasts and a development of new bone in the vacated space through the agency of the osteoblasts.

In a case of compound, comminuted fracture inflammation results and the various changes take place, the first effort towards recovery being made in the soft tissues. The exudate and useless portions of bone are thrown off by suppuration. The irritation of the periosteum and medulla excites a proliferation of the osteogenetic cells, and the ring and pin callus are formed for the purpose of keeping the parts immovable during the next stage, when the true bony union takes place between the ends of the fragments. This being accomplished the osteoclasts absorb the now useless natural splints and the bone is fully repaired.

In ranula, where the calculus is too large to pass through the duct, obstruction occurs and inflammation results in an abscess and the stone escapes through the fistula.

Compensation for loss or impairment of a part cannot better be illustrated than by citing the hypertrophy of the left ventricle of the heart when aortic stenosis or arterio-capillary fibrosis renders more difficult the keeping up of the circulation.

When an artery is tied there results a diminished supply of blood to the part affected, but, as Black says, the nerves soon take cognizance of the fact that the tissues are not getting their proper nutrition and vasomotor impulses stimulate an increased supply through the anastomosing branches.

When an artery is cut and its branches towards the periphery become useless, or when new tissue is forming, there spring from the existing blood-vessels buds which elongate into fibres and unite with each other, finally developing into capillaries as corpuscles are forced through them.

One of the best examples of organization is the isolation of a bullet in the tissues by surrounding it with a sheath of dense fibrous tissue, rendering its presence harmless. Two physiological actions are finely illustrative of the compensative power of nature. One is the absorption of the roots of the temporary teeth and their retention until the permanent ones are about ready to come through. The other is the multiplication in size and number of the cells of the uterus during pregnancy and the return to normal after delivery.

Every action has its purpose and explanation, and close minute study reveals the secret workings of nature in her efforts to rebuild and repair the damage done by accident and disease. She works with reason and consistency, and whatever aid is offered must be given with the same reason and consistency and with a thorough knowledge of the pathological actions of cells and tissues.

